# Heterogeneity and high prevalence of bone manifestations, and bone mineral density in congenital generalized lipodystrophy subtypes 1 and 2

**DOI:** 10.3389/fendo.2024.1326700

**Published:** 2024-04-03

**Authors:** Erika Bastos Lima Freire, Catarina Brasil d’Alva, Mayara Ponte Madeira, Grayce Ellen da Cruz Paiva Lima, Virginia Oliveira Fernandes, Lindenberg Barbosa Aguiar, Leonardo Barreira Portella, Renan Galvão Ozório, Clarisse Mourão Melo Ponte, Ana Paula Dias Rangel Montenegro, Renan Magalhães Montenegro Junior

**Affiliations:** ^1^ Brazilian Group for the Study of Inherited and Acquired Lipodystrophies (BRAZLIPO), Fortaleza, Brazil; ^2^ Clinical Research Unit, Walter Cantídio University Hospital, Federal University of Ceará/EBSERH, Fortaleza, CE, Brazil; ^3^ Department of Clinical Medicine, Federal University of Ceará, Fortaleza, CE, Brazil; ^4^ University of Fortaleza, (UNIFOR), Fortaleza, CE, Brazil; ^5^ Department of Community Health, Federal University of Ceará, Fortaleza, CE, Brazil; ^6^ Radiology Unit, Walter Cantídio University Hospital, Federal University of Ceará/EBSERH, Fortaleza, CE, Brazil; ^7^ Diagnostics of America (DASA), São Paulo, SP, Brazil; ^8^ Christus University Center, Fortaleza, CE, Brazil; ^9^ Pediatric Endocrinology Unit, Walter Cantídio University Hospital, Federal University of Ceará/EBSERH, Fortaleza, CE, Brazil

**Keywords:** Berardinelli-Seip syndrome, bone, lipodystrophy, bone mineral density, bone cyst

## Abstract

**Introduction:**

Congenital Generalized Lipodystrophy (CGL) is a rare autosomal recessive disease caused by mutations in genes responsible for the formation and development of adipocytes. Bone abnormalities are described. However, there is a scarcity of data.

**Objective:**

To describe bone characteristics in a large CGL1 and 2 case series.

**Methods:**

Cross-sectional study that assessed bone radiological features of CGL patients of a reference hospital in Fortaleza (CE), Brazil. Patients underwent clinical and bone mineral metabolism evaluation, radiographs of the axial and appendicular skeleton and bone mineral density (BMD) assessment by DEXA (dual energy X-ray absorptiometry).

**Results:**

Nineteen patients were included, fourteen were CGL1 and 5, CGL2. Median age was 20 years (8–42) and 58% were women. Median BMI and percentage of body fat were, respectively, 21 Kg/m² (16–24), and 10.5% (7.6-15). The median leptin concentration was 1 ng/mL (0.1-3.3). Diabetes mellitus and dyslipidemia were present in 79% and 63% of patients, respectively. Median calcium and phosphate were normal in almost all patients (95%). Median parathyroid hormone and 25-OH-vitamin D were 23 pg/mL (7-75) and 28 ng/mL (18-43). Osteolytic lesions, osteosclerosis and pseudo-osteopoikylosis, were present in 74%, 42% and 32% of patients, respectively. Lytic lesions were found predominantly in the extremities of long bones, bilaterally and symmetrically, spine was spared. Osteosclerosis was present in axial and appendicular skeleton. Pseudo-osteopoikilosis was found symmetrically in epiphyses of femur and humerus, in addition to the pelvis. BMD Z-score greater than +2.5 SD was observed in 13 patients (68.4%). BMD was higher in CGL1 compared to CGL2 in lumbar spine and total body in adults. No associations were found between high BMD and HOMA-IR (p=0.686), DM (p=0.750), osteosclerosis (p=0.127) or pseudo-osteopoikilosis (p=0.342), and, between pain and bone lesions. Fractures were found in 3 patients.

**Conclusion:**

Bone manifestations are prevalent, heterogeneous, and silent in CGL1 and CGL2. Osteolytic lesions are the most common, followed by osteosclerosis and pseudo-osteopoikilosis. Bone mass is high in most cases. There was no pain complaint related to bone lesions. Thus, systematic assessment of bone manifestations in CGL is essential. Studies are needed to better understand its pathogenesis and clinical consequences.

## 1 Introduction

Congenital Generalized Lipodystrophy (CGL) is an autosomal recessive disease caused by mutations in genes responsible for the formation and development of adipocytes, resulting in total or almost total loss of adipose tissue with consequent ectopic accumulation of lipids (1–3). Therefore, patients develop insulin resistance and severe metabolic disorders such as diabetes mellitus (DM), dyslipidaemia and nonalcoholic fatty liver disease (1, 4). They seem to have high cardiovascular risk due to the high prevalence of these metabolic diseases, although atherosclerotic vascular complications have only been described in a few patients (5). Most patient evolve with cardiomyopathy, which can contribute to their reduced life expectancy (6). Death is usually caused by liver and diabetic complications (renal failure and sudden death) in addition to infections (7). The disease is classified into four subtypes according to the mutated gene*: ;AGPAT2, BSCL2, CAV1 and ; CAVIN1* (1, 8) and approximately 95% of patients currently described harbor mutation in *AGPAT2* (CGL1) or *BSCL2* (CGL2). These two subtypes can be differentiated according to their clinical presentation since mechanical adipose tissue is spared in CGL1. Absence of mechanical fat is very suggestive of CGL2 (9). Another clinical feature that has been used to distinguish CGL1 and CGL2 is the presence of bone cysts, which has been considered, by some authors, to be specific of CGL1 (10).

Subtypes 1 and 2 present no bone marrow adipose tissue, in contrast to the less common subtypes of CGL (caused by mutations *in CAV1-*CGL3 and *CAVIN-*CGL4) who have preserved medullary fat (11). Therefore, the mutations have distinct effects on different types of adipose tissue, which could justify, in part, the clinical heterogeneity of this syndrome.

There are few studies on bone radiological findings in CGL, apart CGL4 (12). Most are case reports or series of cases (1). Recently, Teboul-Coré et al. (13) described three main types of bone abnormalities in CGL: diffuse osteosclerosis, lytic lesions, and pseudo-osteopoikilosis. Regarding bone mineral density (BMD), Lima et al. (14) studied 21 CGL patients and found high BMD Z-score (Z-score greater than +2.5 SD) in most individuals, regardless of the presence of conditions that negatively influence bone mass.

In this study, we describe bone radiographic characteristics and BMD Z-score of CGL patients from a Brazilian large case series.

## 2 Materials and methods

### 2.1 Study design and participants

This is a cross-sectional study in which all patients with CGL diagnosis enrolled at the Endocrinology Service in the university hospital of the Universidade Federal do Ceará (UFC)/EBSERH were invited to participate. This hospital, which is head office of the Brazilian Group for the Study of Inherited and Acquired Lipodystrophies (BRAZLIPO), is a reference center on the care of lipodystrophy patients in the state of Ceará. The main inclusion criterion was the clinical diagnosis of CGL according to PATNI and GARG (1). Molecular analysis confirmed the CGL diagnosis in all subjects. Exclusion criteria were refusal to participate and chronic kidney disease, as this condition can also lead to osteosclerosis and may represent a confounding factor (15). This study was approved by the Institutional Ethics Committee of our university hospital (protocol number: 4.564.593). Informed written consent was obtained from all adult participants and parents or guardians for participants under the age of 18.

### 2.2 Data collection

All individuals underwent medical evaluation to obtain the following data: gender, age, body mass index (BMI), diagnosis of DM, dyslipidemia, history of previous bone fractures and their mechanisms, as well as bone pain. The medical records of all patients were reviewed to complement data obtained during the interview.

Total cholesterol (TC), HDL cholesterol, triglycerides (TG), and blood glucose were obtained after overnight fast and determined according to standard methods using automated equipment. Serum concentrations of total calcium, phosphorus, and albumin were determined on an automatic biochemical analyzer (CMD 800ix1, Wiener Lab Group, Argentina). Calcium and phosphate in adults and children had respective reference ranges: 8.3-10.5 mg/dL, 2.5-5.6 mg/dL and 4.0-7.0 mg/dL, and calcium was adjusted for albumin concentration. Serum parathyroid hormone (PTH) concentrations were measured by chemiluminescence (IMMULITE 2000, Siemens, Llanberis, United Kingdom). PTH normal range was 11-67 pg/mL, intra-assay and inter-assay coefficients of variation were 5.7% and 8.8%, respectively. Glycated hemoglobin (A1c) values were determined by ion exchange high-performance liquid chromatography (HPLC) and serum basal insulin levels (in non-insulin users) were measured by immunoassays with reagents provided by Roche Diagnostics (Basel, Switzerland). HOMA-IR (homeotase evaluation model = fasting glucose [mmol/L] × fasting insulin [μU/mL]/22.5) higher than 2.7 was indicative of insulin-resistance in non-insulin users (16, 17). Serum 25-OH-vitamin D (25OHD) was determined by chemiluminescence (Architect, Abbott, Sligo, Ireland), with intra-assay and inter-assay coefficients of variation 2.9 and 5.5%. Adequate total body vitamin D stores were defined at a serum 25OHD level ≥ 30 ng/mL and 25OHD insufficiency was considered when levels were below 30 ng/mL (18).

All patients underwent radiography of the axial and appendicular skeleton and had BMD assessed by DEXA (dual energy X-ray absorptiometry, Ge Prodigy Lunar). The absolute value of BMD was measured in g/cm^2^ in the lumbar spine (L1-L4), femoral neck (FN), total proximal femur (TPF) and total body (TB) in adults and in the L1-L4 and total body less head (TBLH) in children (19). BMD was compared with a reference database of individuals of the same age and gender to obtain the Z-score, calculated in standard deviations (SD), since all individuals were under 50 years old. Low BMD was considered when Z-score was less than -2.0 SD and, high BMD when Z-score was greater than + 2.5 SD (14, 20).

In the axial skeleton, radiographs of the cervical, thoracic, and lumbar spines, as well as pelvis were obtained in anteroposterior (AP) and lateral incidences. In the appendicular skeleton, hand radiographs were performed in posteroanterior (PA) incidence, feet in AP incidence, upper and lower limbs in AP and lateral incidences.

All radiographs were analyzed by two radiologists, one of them with experience in CGL radiological features, and one BRAZLIPO endocrinologist. Radiographs were reviewed for three main bone findings: osteolytic lesions, diffuse osteosclerosis and pseudo-osteopoikilosis ( ;multiple patchy sclerotic bone islands) and their distribution among bone segments (diaphysis, metaphysis, or epiphysis of long bones). We also looked for radiographic signs of fracture.

### 2.3 Statistical analysis

The numerical variables were presented at median (minimum-maximum). Categorical variables were described in frequency and prevalence. Mann-Whitney U test was used to analyze the characteristics of the participants, verifying non-adherence of the data to the Gaussian distribution. For the analysis of categorical variables, we used Pearson’s chi-square test and Fisher’s exact test. A significance level of 5% was adopted. Statistical analyses were performed using the JAMOVI statistical program and Microsoft Excel 2016.

## 3 Results

### 3.1 Sample description

At the time of this study, 22 patients with stablished CGL diagnosis were registered at the Endocrinology section of our institution and 19 were included. One patient was excluded due to chronic kidney disease and two refused to participate. Eleven individuals (58%) were women. Molecular analysis identified that 14 patients (74%) were CGL1 while 5 (26%), CGL2. The median age was 20 years (8-42 years). Three patients (16%) were younger than 10 years old, six (31%) were between 10 and 19, and ten (53%) were older than 20.Median BMI and percentage of body fat were, respectively, 21 Kg/m² (16-24 kg/m^2^), and 10.5% (7.6-15%.) The median leptin concentrations were 1 ng/mL (0.1-3.3 ng/mL) (**Table 1**).

**Table 1:** Clinical and biochemical characteristics of patients with Congenital Generalized Lipodistrophy.

	CGL1	CGL2	TOTAL	*p*
**N (% female)**	14 (57%)	5 (60%)	19 (58%)	>0.999
**Age (yr)**	22 (6–41)	16 (8-34)	19 (6-41)	0.754
**BMI (kg/m^2^)**	22 (16 -24)	19 (16-24)	21 (16.-24)	0.331
**Body fat (%)**	11.2 (8.1-15)	8.7 (7.6-9.1)	10.5 (7.6-15)	**0.018**
**Leptin (ng/mL)**	1 (0.1-3.3)	1 (0.1-1.4)	1 (0.1-3.3)	0.709
**Diabetes n (%)**	11 (79%)	4 (80%)	15 (79%)	>0.999
**Insulin therapy n (%)**	8 (57%)	3 (60%)	11 (58%)	>0.999
**Metformin use n (%)**	13 (93%)	5 (100%)	18 (95%)	>0.999
**Statin use**	4 (29%)	2 (40%)	6 (32%)	>0.999
**Fibrate use**	3 (21%)	0 (0%)	3 (16%)	0.530
**A1c %**	8.2 (4.5-12.5)	8.3 (5.6-11)	8.3 (4.5-12.5)	0.687
**HOMA-IR**	6.0 (1.9-47.3)	12.1 (3.8-20.3)	6.3 (3.8-47.3)	0.859

Leptin reference range 0.5-7.9 ng/mL. Bold values indicate that the value of P is significant (p < 0.05).

Regarding the glycemic profile, 15 patients (79%) had DM and 11 (58%) were on insulin therapy. Eighteen individuals (95%) were on metformin. Despite of this medication use, the eight (42%) non-insulinized patients presented median HOMA-IR of 6.3 (1.9-47.3). Only one patient had its value below 2.7 (1.9). The median A1c considering diabetics and non-diabetics was 8.5% (4.5-12.5%) and 72% of patients had it above 7.0%. Regarding the lipid profile, the median triglycerides was 169 (61-1800 mg/dL) and 63% of patients had values above 150 mg/dL. The median HDL was 29 mg/dL (23-62 mg/dL). Only six (32%) patients were on statin therapy and 3 (16%) on fibrate.

The evaluation of bone mineral metabolism revealed total calcium corrected by albumin 9.5 mg/dL (8.3-10.1 mg/dL). Calcium and phosphate serum levels were normal in all patients, except for a patient with postsurgical hypoparathyroidism, due to papillary thyroid carcinoma, also the only one with low PTH. The median PTH value was 23 pg/mL (7-75 pg/mL). Only one patient, with a 25OHD of 26 ng/mL, had elevated PTH (75 pg/mL). The median 25OHD was 28 ng/mL in the 18 evaluated patients (15-43 ng/mL). Eleven patients (61%) had 25OHD levels between 20-30 ng/mL and only one below 20 ng/mL. No patients were taking vitamin D supplements. All adults had normal alkaline phosphatase level, median 185 U/L (92-301 U/L), however it was increased in 5 (56%) of patients younger than 20 years of age, median 700 U/L (340 -1157 U/L) (Individual general characteristics, metabolic profile and evaluation of bone metabolism are arranged in **Appendix 1**).

### 3.2 Bone manifestations

#### 3.2.1 Lytic lesions

**Figure 1:**
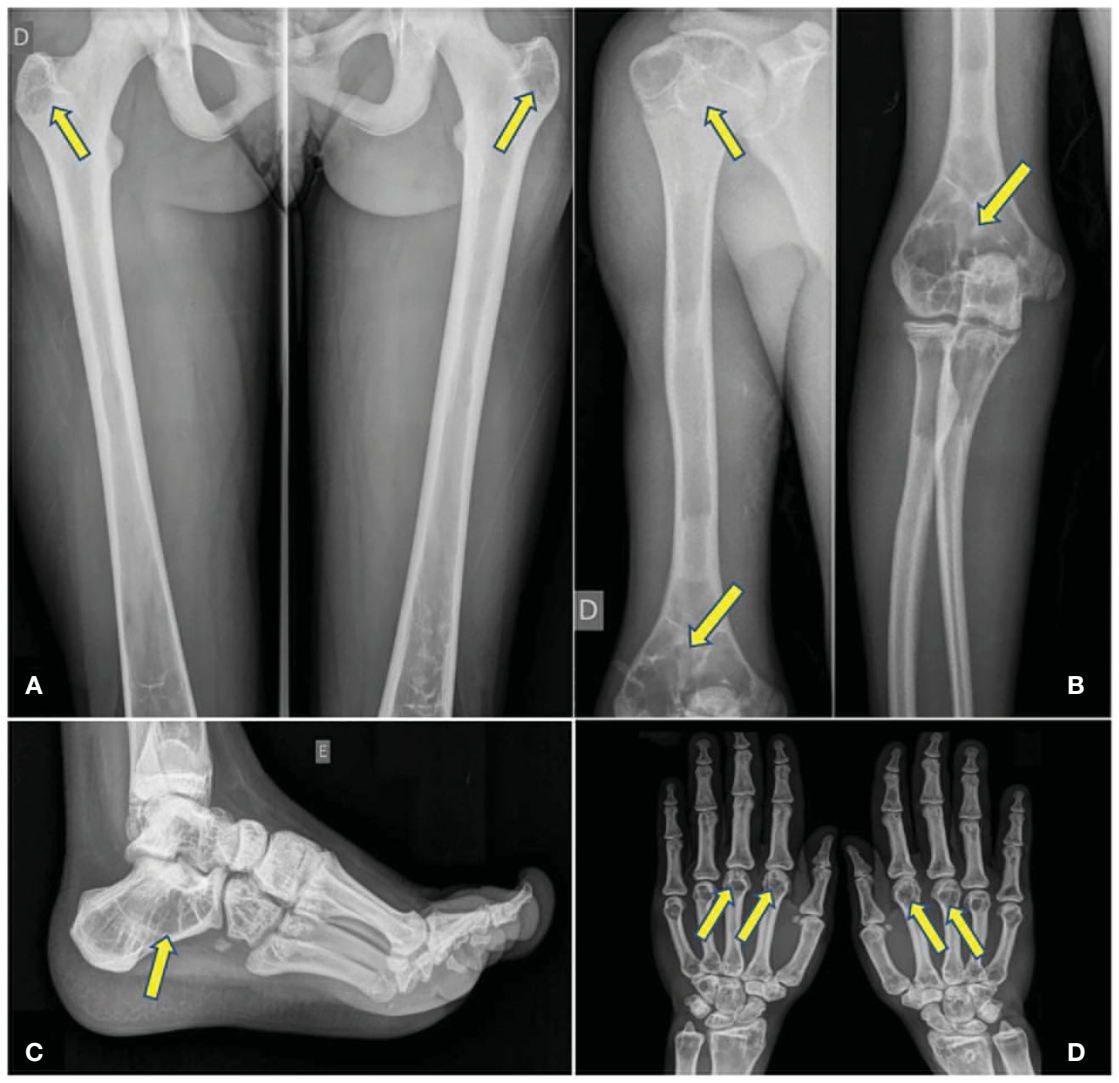
**(A)** Bilateral simple cystic lesions. **(B)** Cystic lesions in metacarpal bones. **(C)** Honeycomb lesions. **(D)** Accentuation of vertical trabeculate in tarsus.

Osteolytic lesions were categorized into simple (single or isolated, cystic or not) (**Figures 1A, D**) and multicystic (honeycomb style) lesions (**Figure 1B**). In addition, areas of accentuation of the vertical bone trabeculate due to intermediate osteolytic areas were observed (**Figure 1C**).

Single or multiple osteolytic lesions were present in 14 (74%) patients, being 12 CGL1 (86%) and 2 CGL2 (40%). They were observed in long bones, hands, and feet. The spine was spared in all patients.

A total of 138 single or multicystic lesions (129 in 12 CGL1 and 9 in 2 CGL2) were found in the following bones: femur, feet, tibia, humerus, hands, fibula, radio, ulna and achromy. Femur was the most common site of lytic lesions. Only 20 (14%) of the 138 lesions were unilateral and were found in 10 (57%) patients (8 CGL1 and 2 CGL2). Therefore, 118 lesions (86%), 59 pairs, had bilateral and symmetrical involvement (**Table 2**).

**Table 2:** Number of patients with CGL1 and CGL2 with simple or multicystic lytic lesions in different bone sites.

	CGL1 (n =14)			CGL2 (n =5)			TOTAL (n =19)
	Simple	Multicystic	Simple or multicystic*(%)	Simple	Multicystic	Simple or multciystic*(%)	
**Femur**	7	2	9 (64%)	1	0	1 (20%)	10 (53%)
**Feet**	7	1	7 (50%)	1	2	2 (40%)	9 (47%)
**Tibia**	8	0	8 (57%)	1	0	1 (20%)	9 (47%)
**Hands**	7	0	7 (50%)	1	0	1 (20%)	8 (42%)
**Humerus**	5	6	8 (57%)	0	0	0	8 (42%)
**Radio**	5	1	5 (36%)	0	0	0	5 (26%)
**Ulna**	4	1	5 (36%)	0	0	0	5 (26%)
**Fibula**	2	0	2 (14%)	0	0	0	2 (10%)
**Achromy**	0	1	1 (7%)	0	0	0	1 (5%)

*The total number may not correspond to the sum because some patients have multicystic and simple lesions in the same bone.

Simple lytic lesions, the most common presentation, were found in 13 patients (68%), 12 CGL1 and 1 CGL2. All these individuals presented lesions with clear margins, well defined, without sclerotic edges, although 4 patients (3 CGL1 and 1 CGL2) also presented lesions with sclerotic margins. Multicystic lesions (honeycomb style) were found in 8 individuals (42%), 6 CGL1 and 2 CGL2. These lesions were bilateral and symmetrical in all patients, except in an individual who had suffered a previous amputation of the right foot. Both simple and multicystic lesions were located mainly in the epiphysis and metaphysis of long bones (76%). Diaphysis harbored 24% of the lytic lesions.

Concerning to the age at which cystic lesions appear, in our cohort was composed by 3 children younger than 10 years, 6 patients between 10 and 19 years and 10 patients older than 19 years, multicystic lesions were only present after age 10, but simple cysts were identified in 2/3 CGL1 children younger than 10 (they were not present in the only CGL2 child).

We also observed sites with accentuated vertical bone trabeculate, due to intermediate osteolytic areas in 16 patients (84%), 12 CGL1 and 4 CGL2. Thirteen (68%) patients presented this manifestation in feet (predominantly in tarsus and calcaneus), 4 (21%) in tibia, 3 (16%) in femur, 2 (10%) in fibula and 1 (5%) in humerus. The pattern was bilateral and symmetrical in all patients.

#### 3.2.2 Osteoesclerosis

**Figure 2:**
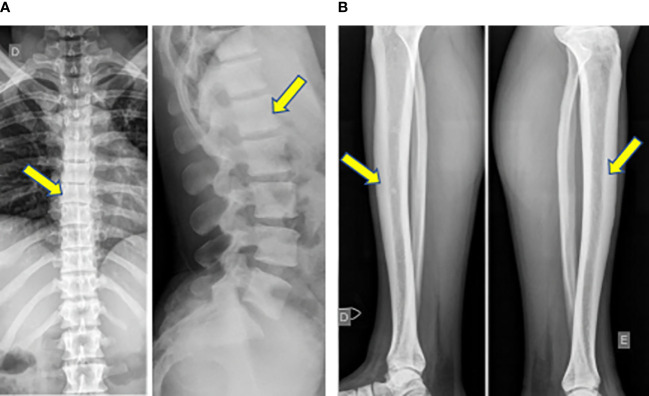
**(A)** Homogeneous osteosclerosis in the spine in a CGL1 patient. **(B)** Diffuse cortical thickening in long bones, resulting in reduction of the medullary cavity in a CGL1 patient.

Osteosclerosis was observed in 8 CGL1 patients (42%) diffusely throughout both in axial and appendicular skeleton. It was homogeneous throughout the spine in all 8 patients (**Figure 2A**). In long bones, the observed pattern was diffuse cortical thickening, present in 6 CGL1 (32%), often resulting in reduction of the medullary cavity (**Figure 2B**). It was not found in individuals with CGL2.

#### 3.2.3 Pseudo-osteopoikilosis

**Figure 3:**
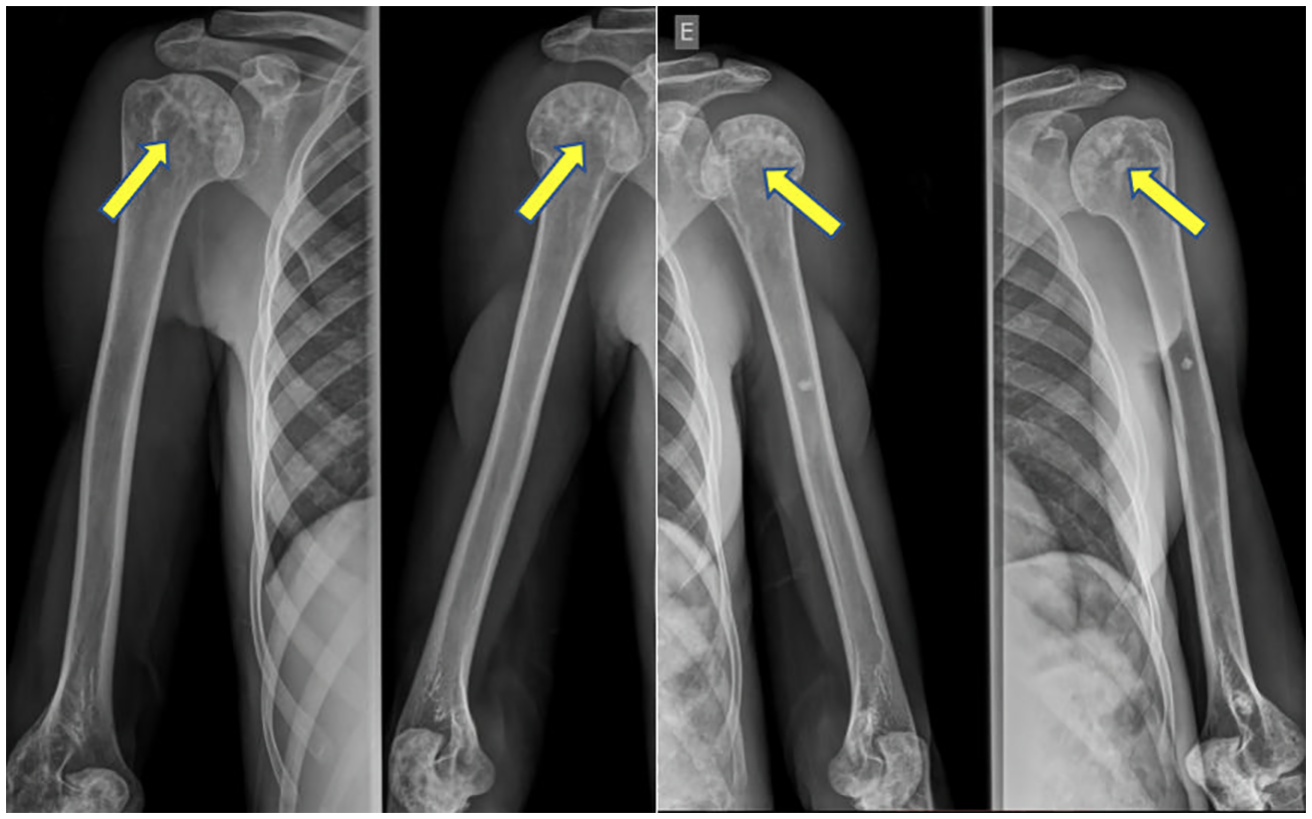
Pseudo-osteopoikilosis in humeral heads bilaterally.

Pseudo-osteopoikilosis was observed in 6 (32%) patients, 4 CGL1 (29%) and 2 CGL2 (40%). It was found in epiphysis of the femur and humerus, bilaterally and symmetrically, in 5 and 2 patients, respectively, and in the pelvis in 4 patients (**Figure 3** **).**

### 3.3 Bone mineral density

Increased BMD Z-score (Z-score greater than +2.5 SD) in at least one bone site was confirmed in 13 patients (68%), 11/14 CGL1 (79%) and 2/5 CGL2 (40%) (**Table 3**). Analyzing the 13 patients with increased BMD Z-score, high bone mass was observed in the TBLH of children or TB of adults in 12/13 individuals (92%) and lumbar spine in 9/13 individuals (69%). Considering only the adults, high BMD Z-score was found in the FN in 4/10 (40%) and in the TPF in 5/10 (50%) individuals (**Table 4**).

**Table 3:** BMD in CGL, CGL1 and CGL2.

	CGL	CGL1	CGL2*p*
**L1-L4 -BMD (g/cm2)**	1.210 (0.720 - 1.760)	1.256 (0.770 -1.760)	1.110 (0.720 - 1.220)
**Z-score**	1.6 (-3.4 to + 4.8)	**2.5** (-0.8 to + 4.8)	-0.2 (-3.4 to + 2.5) **0.046**
**FN BMD (g/cm2)**	1.260 (0.920 - 1.700)	1.338 (0.880 - 1.710)	1.070(0.980 - 1.170)
**Z-score**	1.9 (-1.2 to + 4.6)	**2.5** (-1.2 to + 4.6)	0.1 (-1 to +1.2) 0.400
**TPF BMD (g/cm2)**	1.320 (0.960 - 1.750)	1.356 (0.960- 1.750)	1.030 (1.000 - 1.050)
**Z-score**	2.2 (-0.8 to+ 5.8)	**2.95** (-0.4 to+ 5.8)	-0.15 (-0.8 to +0.5) 0.089
**TB BMD (g/cm2)**	1.360 (1.090 - 1.650)	1.412 (1.100 - 1.650)	1.120 (1.090 - 1.140)
**Z-score**	2.2 (-0.6 to + 5.2)	**2.65** (+0.2 to + 5.2)	-0.25 (-0.6 to+ 0.1) **0.049**
**TBLH BMD (g/cm2)**	1.010 (0.760 - 1.320)	1.023 (0.800 - 1.320)	1.070 (0.760 - 1.130)
**Z-score**	2.3 (-2.3 to + 5.5)	**2.75** (+0.6 to + 5.5)	**2.6** (-2.3 to + 3.1) 0.604

L1-L4, lumbar spine; FN, femoral neck; TPF, total proximal femur; TB, total body; TBLH, total body less head. Bold and underlined values indicate instances when the Z score is greater than 2.5, which signifies the diagnosis of high bone mass.

**Table 4:** CGL1 and CGL2 -patients with increased BMD Z-scores in different bone sites.

	CGL1 (n=14)		CGL2 (n=5)	TOTAL (n=19)
L1-L4	8 (57%)		1(20%)	9 (47%)
FN	4/10 (40%)		0	4/10(40%)
TPF	5/10 (50%)		0	5/10(50%)
TB or TBLH	10 (71%)		2(100%)	12(63%)

L1-L4, lumbar spine; FN, femoral neck; TPF, total proximal femur; TB, total body; TBLH, total body less head.

The analysis of the BMD Z-score in the two groups revealed significantly higher values in lumbar spine and total body in CGL1 compared to CGL2 (**Table 3**). We didn’t find correlations between age and BMD Z-scores in any evaluated bone site (lumbar spine, femoral neck, total femur and total body in adults and lumbar spine and total body less head in children). We also didn’t find differences in the BMD Z-scores between genders in any evaluated bone sites.

We didn’t find associations between high BMD Z-score and HOMA-IR (p=0.686), presence of DM (p = 0.750), osteosclerosis (p = 0.127) and pseudo-osteopoikilosis (p = 0.342). Only one 16-year-old hypogonadal CGL2 patient with liver cirrhosis and low BMI had low BMD Z-score.

### 3.4 Other radiologic findings

**Figure 4:**
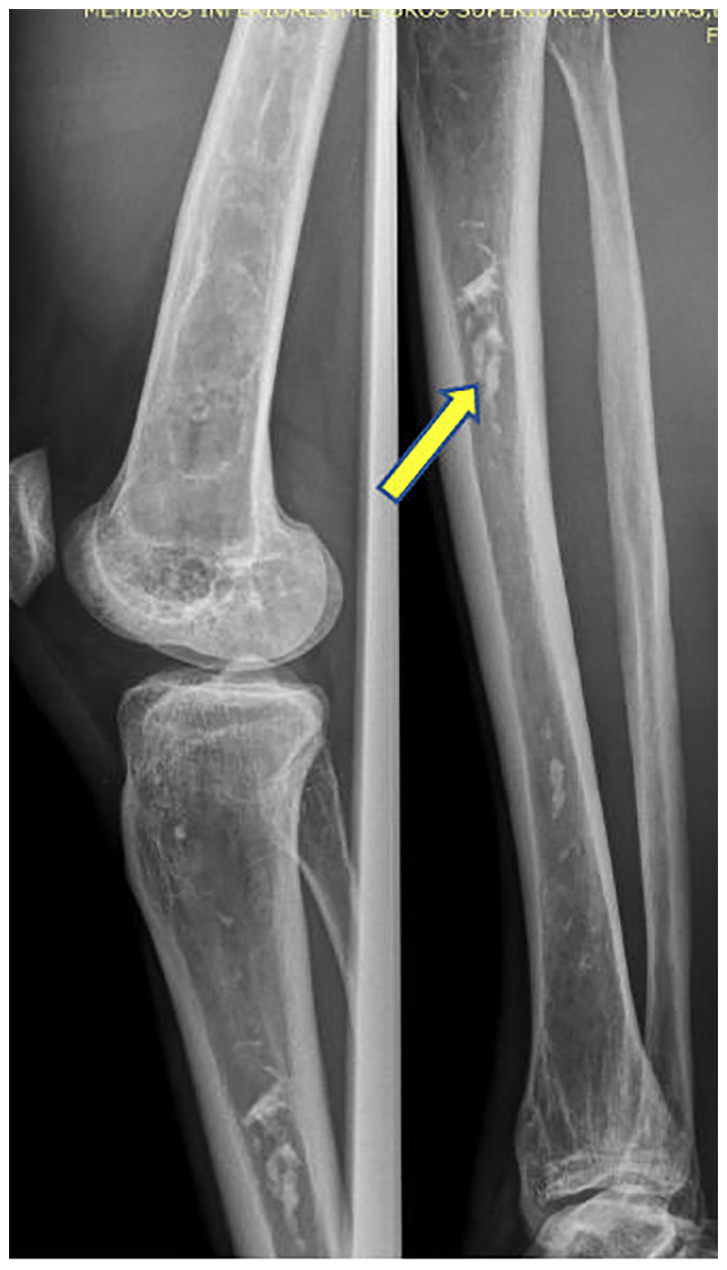
Intramedullary Serpinginous Calcifications.

Five patients (26%) presented serpinginous calcifications in the medullary cavity, consistent with localized osteonecrosis or bone infarction, all CGL1 (**Figure 4**). It was not found in CGL2.

### 3.5 Clinical findings

Regarding the possible clinical consequences of bone lesions, of the total of 19 patients, only three (16%) complained of pain, however, none of them were related to lytic lesions.

### 3.6 Fractures

Three patients (16%) had a history of fracture (two traumatic clinical fractures and one non-traumatic morphometric fracture), all with CGL1. Only one of these patients had fractures in sites of lytic lesions (wrists and shoulder, and soon after right wrist again), although they were caused by a motorcycle accident. Only in a 32-year-old CGL1 woman, who denied pain or trauma, a morphometric bone fracture was identified on radiological examination characterized by a 25% reduction in the anterior height of the vertebral body of T7, which characterized a mild fracture. She presented diffuse osteosclerosis with cortical thickening and high BMD (Z-score +4.4 in L1-L4 and +4.4 in FN).

## 4 Discussion

We report bone radiographic and densitometric findings of a significant series of CGL patients. As far as we know, for the first time, patients underwent an extensive bone radiological inventory, and this is the largest study of radiological features in this disease.

Bone findings rarely lead to the diagnosis of CGL. However, the knowledge of skeletal features of this rare syndrome could help to establish the diagnosis in less symptomatic patients (7). In addition, radiological features of CGL are similar to other bone conditions. In the past, bone findings have led to an incorrect diagnosis of systemic cystic angiomatosis (2, 21). Recently, our group reported a case of a 42-year-old CGL1 man who was misdiagnosed with Paget’s disease prior to the diagnosis of CGL (22). Bone radiological findings, especially osteosclerosis with cortical thickening, may be present in both diseases, which makes clinical correlation very important.

As CGL is a rare disease, there are very few studies on its bone radiological findings. In 2014, Scheller et al. (11) characterized the bone phenotype of two subgroups of CGL patients; the first group composed of CGL1,CGL2 and individuals with unknown mutation (n=75), and the other group,CGL3 and CGL patients (n=18). In the first group, advanced bone age and accelerated bone growth were observed in the first decade of life followed by cortical thickening of the long bones and osteosclerosis in the axial skeleton. After the first decade of life, some CGL1 and CGL2 subjects developed progressive cystic lesions in appendicular skeleton with preservation of bone mass, as well as osteosclerosis. The second group did not present advanced bone age, accelerated bone growth, osteosclerosis or bone cysts after puberty. Besides, a low bone mass was verified after the first decade of life.

In 2016, Teboul et al. (13) published bone radiological features of lipodystrophy from a cohort of 10 CGL patients ( ;8 CGL1 and 2 CGL2*)*. The main bone abnormalities identified were lytic lesions, osteosclerosis and pseudo-osteopoikilosis. Bilateral and symmetrical lytic lesions (single or multiple) were the most frequent feature, observed throughout the appendicular skeleton in 80% of patients (7 CGL1 and 1 CGL2). Multicystic-looking lesions were found 5 CGL1 patients. Diffuse osteosclerosis was present in 70% of patients (6 CGL1 and 1 CGL2) in axial and appendicular skeleton. Pseudo-osteopoikilosis was manifested by 40% of patients (only CGL1), most often in the epiphysis and proximal metaphysis of the femurs and iliac bones.

Interestingly, we had a similar proportion of patients with CGL1 and CGL2.Furthermore, as identified by Teboul et al. (13), simple or multicystic lytic lesions were the most frequent bone feature (74% of our patients), characteristically bilateral and symmetrical, observed in epiphysis and metaphysis of long bones and extremities.These findings suggest that lytic lesions are common manifestations in CGL1 and CGL2 in contrast to Van Maldergem et al. (23) who evaluated 70 CGL patients and found bone cysts in only 28% CGL1 and 7% CGL2. The authors considered this bone lesion a rare CGL feature and since then, cystic bone lesions have been more often attributed to CGL1, which according to our study is inappropriate since they are also present in CGL2.

Diffuse bone sclerosis was the second most frequent bone feature in our series, observed in 8 CGL1 (42%). As described earlier, it was homogeneous throughout the axial skeleton and represented by cortical thickening in long bones. Teboul et al. (13) found it in 70% of patients, including a CGL2 patient (13). This discrepancy can be attributed to the difficulty in standardizing the diagnosis of osteosclerosis using radiography.

Pseudo-osteopokilosis was observed in 6 (32%) patients. The proportion was similar to that previously described (40%) by Teboul et al. (9), but in our series it was also found in CGL2, as reported by Yamamoto (24).

Our data on BMD is in agreement with the largest study on BMD in CGL to date, although the majority of patients (71%) in that study had CGL2 (15 CGL2, 3 CGL1, 3 individuals without established genotype) as opposed to our sample of 74% CGL1 (14). The authors found that most individuals (57%) had high BMD (Z-score ≥ +2.5 SD) in at least one bone site, regardless of the presence of conditions that negatively influence BMD (delayed menarche, low vitamin D levels and calcium intake, low BMI, and physical inactivity). The sites with the highest bone mass were the ultradistal radius (not evaluated in our research) and lumbar spine. In our study, increased BMD Z-score in at least one bone site was found in 68% of individuals although most patients had hypovitaminosis D. In the CGL1 group, 79% had high BMD Z-scores and the CGL2 group, 40%. Significantly higher BMD Z-scores were found in lumbar spine and total body in CGL1 compared to CGL2 patients. We also observed a trend towards higher BMD Z-scores in the other bone sites (femoral neck and total proximal femur) in our sample (71% CGL1) compared to the sample of Lima et al. (74% CGL2) (14). Such comparison reinforces the possibility of higher bone mass in CGL1. Interestingly, none of the 3 CGL1 patients from Lima et al. (Lima et al., 2016b) had increased bone mass (14), and they had low BMD Z-score in TBLH. Lima et al. (14) reported 2 CGL2 (9%) patients with low BMD and we observed this finding in only 1 individual (5%); a 16-year-old woman with delayed menarche, liver cirrhosis and low BMI, all known risk factors for low bone mass.

We did not find association between high BMD Z-score and osteosclerosis, although both are different ways of assessing the increase in bone mass. This may be due to the small sample size.

Lima et al. (14) attributed the high BMD to hyperinsulinemia, since, in that study, HOMA-IR was positively correlated with the BMD of all bone sites analyzed, except radio 33%. In our series, we could not establish a statistical correlation between BMD Z-score and HOMA-IR. Besides, if hyperinsulinemia were a determining factor, patients with CGL2, who have a more severe metabolic condition, would have higher bone mass, which was not verified in our sample. In addition, patients with CGL3 and 4, also insulin resistant, would have high bone mass which has not been demonstrated in the literature to date (11).

We did not routinely measure 33% radius, since it is a frequent site of bone cysts that leads to local reduction of bone mass and a misdiagnosis of osteoporosis. Besides, it is usually reserved for cases of hyperparathyroidism, severe obesity or cases in which the spine or hip cannot be measured or interpreted (25).

The pathophysiological mechanism underlying bone lesions and high bone mass in CGL1 and CGL2 is not well understood. Since the first description of bone cysts in 1968, it has been observed after puberty (2). In 1992, ; for the first time, Fleckenstein et al. (26), evaluated bone abnormalities by magnetic resonance imaging (MR) in patients with CGL and found diffuse absence of bone marrow fat. In 1995, the histological analysis of cystic lesions revealed that they are filled with capillaries and thin-walled veins, meaning that the vascularity was abnormal (27). The authors hypothesized that disturbances of the normal physiological process in the conversion of red to yellow marrow could be involved in the physio pathogeny of the lesions, since this gradual process of conversion doesn’t occur in these patients and the appearance of bone cysts occur after puberty. Little has been added to the initial hypothesis (11). This could be reinforced since CGL3 and CGL4, who have preserved medullary fat, have no evidence of bone cysts described so far (11, 13).

The physiological replacement of the red to yellow medulla usually begins shortly before birth, starting in the distal appendicular skeleton (specifically terminal phalanges of feet and hands) towards proximal bones, occurring more rapidly in distal areas (28, 29). At the age seven, medullary fat ; is already detected in the distal epiphysis of long bones and, at age 12, it is observed in diaphysis (28). In the appendicular skeleton, conversion occurs up to age 25 from distal to proximal regions. On the other hand, axial bone conversion continues until advanced age (11). During this physiological process, intramedullary vascularization also changes. Arteries and sinusoids are replaced ; by sparse capillaries, venulas, and thin-walled veins (28).

The fact that red medulla replacement usually begins in the early years in the distal appendicular skeleton (feet and hands) in a centripetal pattern could justify our finding that the accentuation of the vertical bone trabeculate due to intermediate osteolytic areas are present in tarsus and calcaneus in most patients (28). The feet were the second most affected bone site with lytic lesions. In fact, from adolescence on, all patients already had some form of osteolytic involvement. The inability of patients with CGL to replace the red medulla with fat may result in persistence in the hematopoietic tissue of the medulla or its partial replacement by vascular tissue, which, together with the absence of bone marrow fat, could contribute to the formation of osteolytic lesions. Thus, in CGL, arteries and sinusoids may be replaced by sparse capillaries, venulas and thin-walled veins, since the histological evaluation of bone cysts shows cystic areas filled with abnormal vascularization (27). However, this hypothesis of failure in the normal conversion of the red to yellow marrow explaining osteolytic lesions cannot be proved.

Regarding the pathophysiology of the higher BMD in CGL1 and CGL2, the absence of adipose tissue in the bone marrow seems to play a role (11). The lack of fat in the bone marrow would favor an osteoblastic differentiation, since both adipocytes and osteoblasts come from pluripotent mesenchymal stem cells and compete with each other (11, 30). The fact that CGL3 and CGL4, which have normal distribution of bone marrow fat, have low bone mass reinforces the hypothesis (11). Differentiation in osteoblasts or adipocytes is positively regulated by specific transcription factors, such as RUNX2 and PPARy, respectively.

It has been proposed that, under other conditions such as aging and menopause, hormonal disorders (e.g., hypoestrogenism and hypercortisolism) and nutritional scarcity, expansion of medullary fat occurs, with negative influence on osteoblastic differentiation (31). Individual hormonal and nutritional factors can alter medullary transcription factors causing changes in bone mass.

In short, the absence of bone marrow adipose tissue in CGL1 and CGL2 may play a role in the high BMD and osteosclerosis since it may favor osteoblastic differentiation. On the other hand, the maintenance of the hematopoietic medullary tissue or its partial replacement by vascular tissue may be involved, in some way, in the genesis of osteolytic lesions. In our study, we also found a higher BMD Z-score in lumbar spine and total body in CGL1 when compared to CGL2.

Low levels of leptin and adiponectin, as well as hyperinsulinemia have already been implicated in the role of increased bone mass in CGL1 and CGL2 (14, 32). However, insulin resistance is also present in CGL3 and CGL4 without elevated BMD, and studies with adipokines are still controversial. Another possible explanation already reported was the extreme musculature contributing to increased BMD (11, 14).

Three patients (16%) presented non-specific bone pain, not associated with lytic lesions or bone fractures. Although there are some reports of painful bone cysts, our results are in accordance with the scarce literature published so far, in which symptomatic osteolytic lesions are uncommon (2, 13, 24, 33).

Two patients of our study had previous traumatic clinical fractures. Only one patient presented a fragility fracture identified on radiographs. It was a mild morphometric fracture in the thoracic spine, with no history of trauma or seizure, despite a high BMD and the presence of osteosclerosis. This finding could suggest a worse bone quality. There is only one study that assessed the Trabecular Bone Score (TBS) in patients with CGL, a densitometric tool used to evaluate trabecular microarchitecture and fracture risk (34). Among the 11 individuals evaluated (8 CGL2 and 3 CGL1), 8 had normal TBS, showing that most CGL patients have normal bone microarchitecture. Therefore, such fracture in the thoracic spine cannot be explained by bone fragility in these patients.

This is the largest study on radiological features in patients with CGL. All patients underwent molecular genotyping, which led us to adequately compare bone findings in type 1 and 2 CGL, unlike most previous studies.Although our sample consisted mainly of CGL1, with only 5 CGL2, two of these presented cysticlytic lesions. Lytic bone lesions have been considered more prevalent in CGL1 (13, 35). In fact, this finding in the CGL has already pointed to the diagnosis of CGL1 (10). However, our work shows that the presence of bone cysts should not be used in the differentiation between the subtypes of CGL, although they are more frequent in CGL1.

This study has some limitations. First, we only performed radiographic bone evaluation. An investigation with MR and/or CT would be more enlightening. Although this is the largest cohort of bone findings in CGL ever published, it still harbors a small number of cases, especially when it comes to CGL2. Third, we did not study the pathophysiology of bone lesions, which could help to understand the relationships between bone, bone marrow fat and white adipose tissue in this model of extreme insulin resistance.

## 5 Conclusion

Bone manifestations in CGL1 and CGL2 are highly prevalent, heterogeneous, and silent. Osteolytic lesions are the most frequent lesions, primarily in CGL1. They were found primarily in long bones, hands, and feet in a bilateral and symmetrical pattern in bone extremities. The femur was the most affected site. The spine was spared. Although these skeletal manifestations are more frequent in CGL1, almost half of the CGL2 patients presented lytic lesions, and it is therefore inappropriate to use this data to clinically distinguish these subtypes when genotyping is not available. Osteosclerosis was the second most common finding and was present in almost half of the patients but was not identified in the CGL2. Pseudo-osteopoikilosis was the third most common finding and had bilateral and symmetrical pattern. BMD Z-score was high in most patients and was higher in CGL1 compared to CGL2 in lumbar spine and total body. There was no pain complaint related to bone lesions. Future studies are needed to better understand its pathogenesis and clinical consequences.

## Data availability statement

The data sets analyzed for this study can be found in the repository of the Federal University of Ceará (https://repositorio.ufc.br/).

## Ethics statement

The studies involving humans were approved by Institutional Ethics Committee of the Universidade Federal do Ceara (protocol number: 4.564.593). The studies were conducted in accordance with the local legislation and institutional requirements. Written informed consent for participation in this study was provided by the participants’ legal guardians/next of kin.

## Author contributions

APDRM: Supervision, Writing – review & editing. CBDA: Writing – review & editing. CMMP: Supervision, Writing – review & editing. EBLF: Data curation, Investigation, Methodology, Writing – original draft, Writing – review & editing. GECPL: Data curation, Investigation, Methodology, Writing – review & editing. LBP: Data curation, Formal analysis, Writing – review & editing. LBA: Data curation, Formal analysis, Writing – review & editing. MPM: Data curation, Investigation, Methodology, Writing – review & editing. RMMJ: Formal Analysis, Funding acquisition, Project administration, Supervision, Writing – review & editing. RGO: Data curation, Writing – review & editing. VOF: Supervision, Writing – review & editing.
