# Supplementary material for: Heterogeneity and high prevalence of bone manifestations, and bone mineral density in congenital generalized lipodystrophy subtypes 1 and 2
Source: Front Endocrinol (Lausanne). 2024 Apr 3;15:1326700. doi: 10.3389/fendo.2024.1326700 (PMC11021684; doi:10.3389/fendo.2024.1326700)
Supplement: Supplementary file 1 [file Table_1.docx]

| Diagnosis | *AGPAT2* | *AGPAT2* | *AGPAT2* | *AGPAT2* | *AGPAT2* | *AGPAT2* | *AGPAT2* | *AGPAT2* | *AGPAT2* | *AGPAT2* | *AGPAT2* | *AGPAT2* | *AGPAT2* | *AGPAT2* | *BSCL2* | *BSCL2* | *BSCL2* | *BSCL2* | *BSCL2* |
| --- | --- | --- | --- | --- | --- | --- | --- | --- | --- | --- | --- | --- | --- | --- | --- | --- | --- | --- | --- |
| Age In Years | 32 | 37 | 41 | 26 | 10 | 11 | 8 | 20 | 8 | 11 | 14 | 24 | 33 | 42 | 35 | 21 | 12 | 9 | 16 |
| Bmi (Kg/M^2^ | 21.4 | 22.8 | 22.7 | 23.6 | 16.9 | 19.1 | 16.1 | 21.8 | 20.3 | 18.5 | 21.9 | 22.8 | 21.1 | 24.1 | 17.7 | 24.2 | 19.8 | 18.7 | 16.4 |
| Height (m) | 1.67 | 1.64 | 1.64 | 1.62 | 1.48 | 1.56 | 1.35 | 1.72 | 1.45 | 1.64 | 1.71 | 1.60 | 1.70 | 1.55 | 1.61 | 1.76 | 1.59 | 1.55 | 1.61 |
| Body Fat (%) | 12.2 | 8.1 | 13.5 | 11.5 | 13.1 | 10.9 | 13.6 | 8.4 | 15 | 10.3 | 13.2 | 10.8 | 8.7 | 10.5 | 8.7 | 9.1 | 8.1 | 8.8 | 7.6 |
| Leptin (ng/dL) | 0.4 | 1.4 | 0.5 | 1.9 | 0.1 | 0.4 | 3.3 | 1.7 | 1.0 | 1.3 | 1 | 1.4 | 0.2 | 0.2 | 0.1 | 0.9 | 1 | 1 | 1.4 |
| DM | + | + | + | + | - | + | - | + | - | + | + | + | + | + | + | + | + | - | + |
| Insulin Therapy | + | + | + | + | - | - | - | + | - | + | - | - | + | + | + | + | - | - | + |
| Metformin Use | + | + | + | + | + | + | + | + | + | + | + | + | + | + | + | + | + | - | + |
| HOMA-IR | - | - | - | - | 3.1 | 8.7 | 3.2 | - | 9.8 | - | 47.3 | 1.9 | - | - | - | - | 20.3 | 3.8 | - |
| A1c (%) | 10.2 | 9.3 | 7.4 | 12.5 | 4.5 | 12.1 | 5.7 | 9 | 5.5 | 9.9 | 7.5 | 7.2 | 9.8 | 6.7 | 8.3 | 11.1 | 8 | 5.6 | 11 |
| Statin Use | + | + | - | - | - | - | - | + | - | - | - | - | - | + | + | - | - | - | + |
| Fibrate Use | + | - | + | - | - | - | - | - | - | - | + | - | - | - | - | - | - | - | - |
| Total Cholesterol (mg/dL) | 163 | 99 | 225 | 105 | 171 | 145 | 139 | 134 | 130 | 244 | 324 | 180 | 161 | 103 | 96 | 196 | 149 | 130 | 144 |
| Triglicerides (mg/dL) | 378 | 167 | 246 | 549 | 72 | 341 | 135 | 135 | 446 | 756 | 1880 | 545 | 97 | 155 | 115 | 61 | 375 | 120 | 169 |
| HDL (mg/dL) | 35 | 28 | 29 | 27 | 37 | 32 | 32 | 26 | 24 | 41 | 26 | 29 | 41 | 23 | 29 | 62 | 33 | 26 | 33 |
| Corrected Calcium (mg/dL) | 9.1 | 9.2 | 9.4 | 10 | 9.4 | 9.8 | 9.4 | 9.7 | 9.6 | 10 | 9.5 | 9.9 | 9.4 | 8.9 | 9.7 | 9.7 | 8.3 | 9.5 | 9.2 |
| 25 OHD (ng/dL) | 26.4 | 92 | 24.7 | 26.9 | 42.6 | 22.1 | 24.7 | 28.7 | 30.2 | 29.7 | 17.8 | 29.6 | 25.6 | 35.5 | 34.4 | 33.5 | 30.7 | 26.1 | 25 |
| PTH (pg/mL) | 17.1 | 23 | 32.8 | 14.5 | 20.1 | 31.7 | 28.4 | 23.7 | 14.3 | 18.5 | 16.5 | 24 | 23.4 | 25.8 | 33.8 | 12.4 | 6.9 | 75.1 | 16.6 |
| Phosphate (mg/dL) | 4.6 | 4.0 | 5.4 | 4.3 | 5.1 | 6.1 | 5.6 | 4.4 | 7.0 | 6.2 | 6.8 | 5.2 | 4.6 | 4.9 | 2.7 | 4.2 | 8.9 | 6.1 | 5.0 |
| Alcaline phosphatase (mg/dl) | 164 | 92 | 152 | 206 | 584 | 1039 | 1157 | 301 | 971 | 990 | 340 | 209 | 217 | 144 | 129 | 236 | 465 | 700 | 387 |
| Multicystic Lesions | - | + | - | + | + | + | - | - | - | - | - | + | + | - | - | + | - | - | + |
| Simple Lesion With Scleric Edge | - | + | - | - | - | - | - | + | - | - | - | + | + | - | - | - | - | - | + |
| Simple Injury Without Scleric Edge | + | + | + | + | + | + | + | + | + | - | + | + | + | - | - | - | - | - | + |
| Accentuation Of The Vertical Trabecculate | + | + | + | + | + | - | + | + | - | + | + | + | + | + | + | + | + | - | - |
| Axial Osteosclerosis | + | - | + | - | - | - | - | + | - | + | + | + | + | + | - | - | - | - | - |
| Pseudo-Osteopoikilosis | - | + | + | - | - | - | - | + | - | - | - | + | - | - | + | - | + | - | - |
| Z-score L1-L4 | 4,8 | 2,5 | 3,6 | -0,8 | 0,7 | 3,2 | 1,3 | 2,6 | 4,4 | 1,2 | 2,7 | 4,7 | -0,6 | 1,3 | -0,6 | -0,2 | 2,5 | 0,8 | -3,4 |
| Z-score FN | 4,4 | 1,1 | 2,4 | -1,2 |  |  |  | 4,2 |  |  |  | 4,6 | 0,9 | 2,6 | 1,2 | -1 |  |  |  |
| Z -score TPF | 5,1 | 2,7 | 1,1 | -0,4 |  |  |  | 3,5 |  |  |  | 5,8 | 1 | 3,2 | 0,5 | -0,8 |  |  |  |
| Z -score TBLH |  |  |  |  | 0,6 | 2,6 | 3,3 |  | 5,5 | 2,9 | 2,1 |  |  |  |  |  | 2,6 | 3,1 | -2,3 |
| Z -score TB | 4,8 | 2,5 | 2,8 | 0,2 |  |  |  | 3,4 |  |  |  | 5,2 | 1,4 | 2,5 | 0,1 | -0,6 |  |  |  |
| Fracture | + | - | + | + | - | - | - | - | - | - | - | - | - | - | - | - | - | - | - |
| Pain | - | + | + | - | - | + | - | - | - | - | - | - | + | - | - | - | + | + | - |

**Abbr:* A1c % - glycated hemoglobin, BMI – Body mass index FG- Fasting Glucose, DM- Diabetes Mellitus, 25OHD- 25 OH vitamin D, PTH- Parathyroid Hormone BMI – Body mass index, L1-L4 - lumbar spine, FN -femoral neck TPF-total proximal femur TB - total body TBLH - total body less head

**Reference range* Alcaline phosphatase: adults 65-300 U/L infants <645 U/L , Calcium :8.3-10.5 mg/dL, Leptin: 0.5-7.9 ng/dL, Phosphate: adults 2.5-5.6 mg/dL infants 4.0-7.0 mg/dL , PTH: 11-67 pg/mL
